# Effects of the Variation in Brain Tissue Mechanical Properties on the Intracranial Response of a 6-Year-Old Child

**DOI:** 10.1155/2015/529729

**Published:** 2015-10-01

**Authors:** Shihai Cui, Haiyan Li, Xiangnan Li, Jesse Ruan

**Affiliations:** ^1^Tianjin University of Science and Technology, Tianjin 300222, China; ^2^Tianjin Key Laboratory of Integrated Design and On-line Monitoring for Light Industry & Food Machinery and Equipment, Tianjin 300222, China; ^3^Shanghai HAIMA Automobile R&D Co., Ltd., Shanghai 201201, China

## Abstract

Brain tissue mechanical properties are of importance to investigate child head injury using finite element (FE) method. However, these properties used in child head FE model normally vary in a large range in published literatures because of the insufficient child cadaver experiments. In this work, a head FE model with detailed anatomical structures is developed from the computed tomography (CT) data of a 6-year-old healthy child head. The effects of brain tissue mechanical properties on traumatic brain response are also analyzed by reconstruction of a head impact on engine hood according to Euro-NCAP testing regulation using FE method. The result showed that the variations of brain tissue mechanical parameters in linear viscoelastic constitutive model had different influences on the intracranial response. Furthermore, the opposite trend was obtained in the predicted shear stress and shear strain of brain tissues caused by the variations of mentioned parameters.

## 1. Introduction

The epidemiological investigations showed that the craniocerebral injury caused by traffic accidents was one of the main reasons for children's death [1]. Children head injury criteria and endurance limits are of great importance to develop head protective device. Head injury experiments using child cadavers are the most effective way to study head injury criteria and endurance limits [2]. However, the absence of child cadaver experiments largely hampers the understanding of children's craniocerebral injury mechanism in consideration of ethical morality. Recently, finite element (FE) method provides a new way to solve these problems by computational mechanics and computer technology [3–7]. Roth et al. [8] created a head FE model of a 6-month-old child including main anatomical features of the skull, tentorium, fontanels, falx, cerebrospinal fluid (CSF), and cerebrum based on computed tomography (CT) data. In this study, the FE model was used to evaluate the traumatic injury under the shaking and impact loading conditions. Roth et al. [9–11] also developed a detailed 3-year-old child head FE model to investigate child head injury criteria. Compared with a scaled adult head FE model, the result showed that it was not accurate when scaling an adult head to obtain child head. Ruan et al. [12] developed a detailed 6-year-old head FE model from CT data and conducted the validation of FE model. Cao et al. [13] further developed a detailed FE 10-year-old head model by using ANSYS ICEM CFD and HYPERMESH code from CT data.

It is well known that the proper brain tissue material constitutive models and accurate material parameters are the key factors of FE method to investigate craniocerebral injury. However, the mechanical properties of brain tissues varied with child age gradually [14, 15]. Furthermore, the mechanical properties used in the FE head models were mainly obtained from animal experiments or scaled data from adults due to the absence of child cadaver experiments, which limited the accuracy of FE method.

In this study, a FE model with more detail anatomical features was developed based on head FE model created by Ruan et al. [12]. The effects of brain tissue mechanical parameters variation on intracranial response were systematically evaluated according to Euro-NCAP testing regulation. The craniocerebral injury mechanism for children was discussed.

## 2. Methods 

### 2.1. FE Model Description

**Figure 1:**
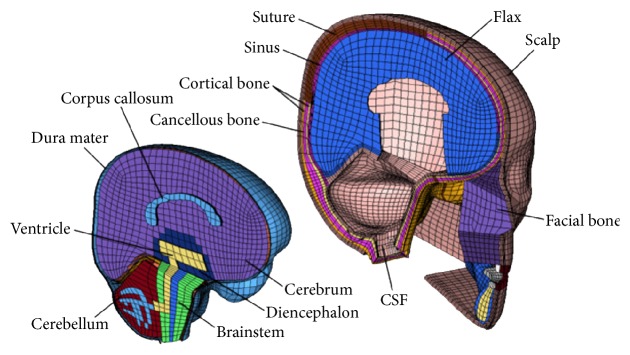
FE model of a 6-year-old child head with detailed head anatomical structures.

Based on the validated FE model created by Ruan et al. [12], intracerebral soft brain tissues were further divided, and hard tissues such as mandibular bones and facial bone were created based on the CT data of a 6-year-old child head using the FE developing method in the literature [21]. Mesh qualities of the FE model were also optimized in this study. The detailed 6-year-old child head FE model was shown in Figure 1; it can be found that the skull includes frontal bone, sphenoid bone, ethmoid bone, the outer plate and inner plate, and undergrown diploe of occipital bone, parietal bones, and temporal bones. The brain soft tissues include cerebrum, corpus callosum, cerebellum, brainstem, ventricle, diencephalon, sinus, flax, CSF, and dura mater. The whole FE head model with 103,716 nodes mainly consisted of 17,346 shells (falx, dura mater, and tentorium) and 96,128 bricks (other brain structures). The meshes among brain tissues, CSF, and skull were connected with common nodes. The developed FE model has been validated in Li's study [22] and Ruan's study [12].

### 2.2. Mechanical Properties of Brain Tissues

The linear viscoelastic constitutive model was commonly applied to investigate brain tissue injury in the head FE models. The Zener model for brain tissues was adopted in this research, and the constitutive equation was defined in the following equation:

(1) $$\begin{array}{c} G\left(\;t\;\right)=G_{\infty }+\left(\;G_{0}-G_{\infty }\;\right)e^{-\beta t}, \end{array}$$

where *G*_*∞*_ is the long-term shear modulus, *G*_0_ is the short-term shear modulus, and *β* is decay coefficient.

Thibault and Margulies [14] compared the linear elastic shear modulus of cerebrum in a 3-day-old piglet (equivalent to a 1-month-old human infant) with that of 1-year-old pig (equivalent to a 4-year-old human infant) and found that the shear modulus was obviously different even at low strains. Chatelin et al. [15] found that child's brain mechanical properties varied evidently with age when children were younger than 2 years under low frequency shear load. The result showed that long-term and short-term shear moduli of white matter, gray matter, and brain stem of children older than 2 years were the same as those of adults. Additionally, the shear modulus of brain stem is two or three times stiffer than that of white matter and gray matter regardless of age. Thibault and Margulies [14] and Dobbing [23] found that the composition of the brain tissue of 2-year-old children, such as DNA polymerase content, water content, and lipid content, was mostly the same as those in adults [9, 11]. The brain mechanical properties in the linear viscoelastic constitutive model reported in the literatures are summarized in Table 1 [9, 16–20].  Table 2 summarizes the other head material properties used in the FE head model.

From Table 1, it can be seen that brain mechanical properties vary in a large range as reported in the literatures. As for the short-term shear models, it varies in the range of 5.99–528 kPa, while the long-term shear modulus varies in the range of 2.32–168 kPa. Decay coefficient varies in the range of 0.09248–920 s^−1^, and *K* varies in the range of 1.25–2110 MPa.

To evaluate the effects of brain tissue mechanical parameters variation on intracranial response, a comprehensive parametric study was conducted. The simulation matrix with different parametric combinations is shown in Table 3. The parameters reported in the literature [9, 17] were adopted as the baseline experiment, where the short-term shear modulus *G*_0_ was taken as 4.9, 49, and 490 kPa, respectively. Long-term shear modulus *G*_*∞*_ varied as 1.62, 16.2, and 162 kPa, respectively. Decay coefficient *β* varied at the levels of 1.45, 14.5, 145, and 1450/s, and bulk modulus *K* varied at the levels of 21.9, 219, and 2190 MPa. These values can cover all the reported data in Table 1. Particularly, the reported results showed that the bulk modulus of brain tissues was at least 10^5^ times than shear modulus [15]. Therefore, the proportion of bulk modulus to shear modulus in all experiment series should meet the special requirements. The experiment series in Table 3 can meet the special requirements.

### 2.3. Load and Boundary Setup of Impact Simulation Experiments

**Figure 2:**
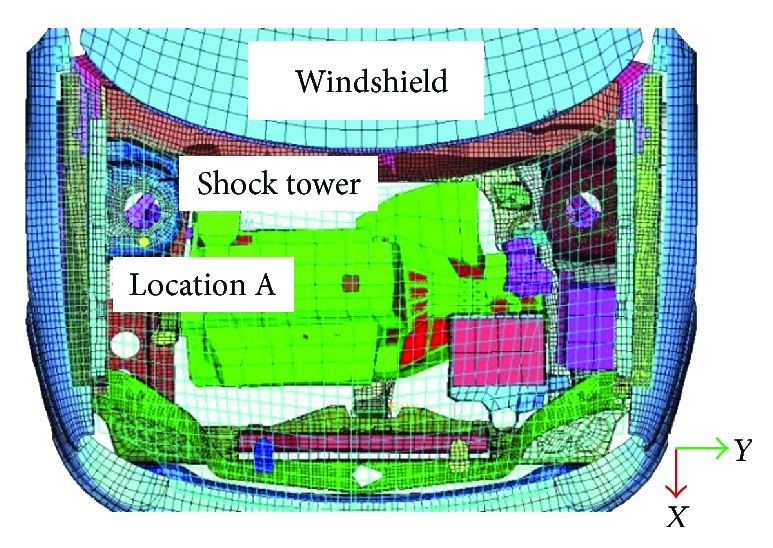
Impact location beneath the engine hood.

The impact between the developed child head model and engine hood was reconstructed according to Euro-NCAP testing regulations [24]. Engine hood surface at location A where injurious structure of shock absorber exists (Figure 2) is selected from child headform test zones as impact location [22].

**Figure 3:**
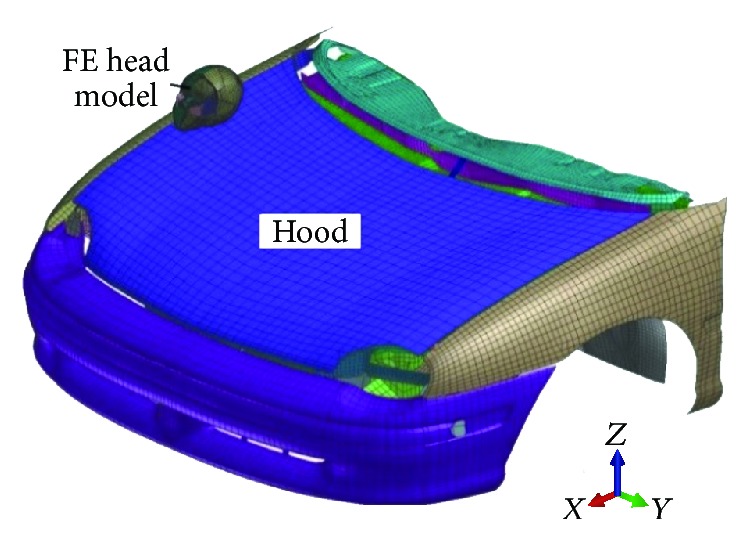
Simulation of impact between FE head model and engine hood.

Figure 3 shows the forehead of FE model impacts location A of the engine hood surface in the simulations, which were conducted by using PAM-CRASH code. The velocity of the center of mass of FE head is set at 35 km/h and the engine hood is still. Velocity direction of FE head was 50 degrees with the horizontal plane, and impact direction was downward and rightward related to front structure on vehicle longitudinal vertical plane.

## 3. Results and Discussion

### 3.1. Intracranial Response with Different *K* Values

#### 3.1.1. Intracranial Pressure

**Figure 4:**
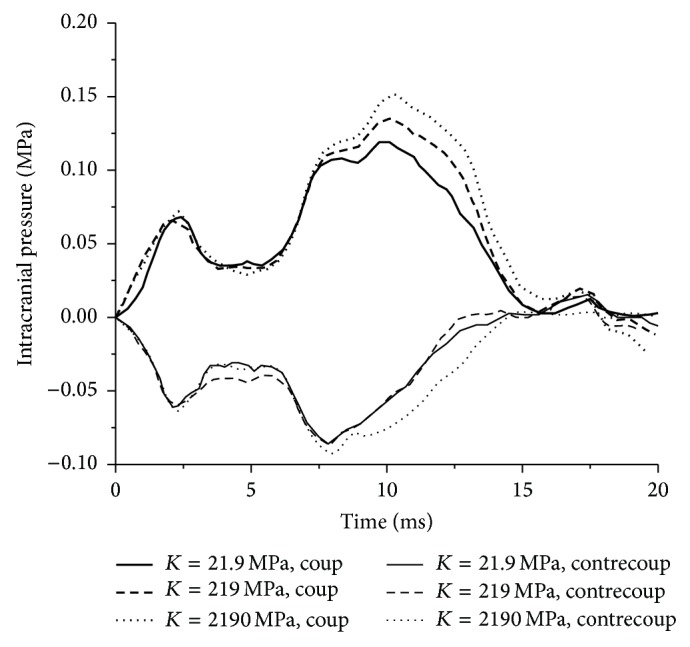
The intracranial pressure time histories with different *K* values.

Though *G*_0_, *G*_*∞*_, and *β* play an important role in intracranial pressure, Ex_1, Ex_6, and Ex_7 experiments in Table 3 only focus on the effect of bulk modulus *K* on intracranial pressure. The results of Ex_1, Ex_6, and Ex_7 are shown in Figure 4 for the intracranial pressure time histories with different *K* values. It shows that both coup pressure and contrecoup pressures decrease with lower *K* values. When *K* value is 21.9 MPa, the peak coup pressure reaches 117.1 kPa and peak contrecoup pressure is −88.1 kPa. The peak coup pressure increases to 159.8 kPa and peak contrecoup pressure is −97.2 kPa, when *K* value increases to 2190 MPa. The decrease of bulk modulus *K* values means that the hydrostatic pressure decreases in that brain tissue. As a result, the brain tissue of the impact side deforms more easily under the same loading conditions, which results in a lower coup pressure accordingly.

#### 3.1.2. Shear Stress and Shear Strain

The peak shear stress increases while shear strain decreases with the increase of *K* value. The peak shear stress increases from 13.7 kPa to 17.9 kPa when *K* value increases from 21.9 MPa to 2190 MPa. However, the shear strain decreases sharply from 0.6 to 0.1. The maximum shear stress occurs at dorsal pontine of brain stem when *K* value is 21.9 MPa and 219 MPa, and it occurs at the dorsal midbrain region of brain stem when *K* value is 2190 MPa. While the maximum shear strain occurs at the lobe of cerebrum parietal cortex in impact side when *K* value is 21.9 MPa, it occurs at ventrolateral pons of brain stem when *K* value is 219 MPa and 2190 MPa. The results mean that location of maximum shear stress is not the same as that of maximum shear strain.

### 3.2. Intracranial Response with Different *G* Values

#### 3.2.1. Intracranial Pressure

**Figure 5:**
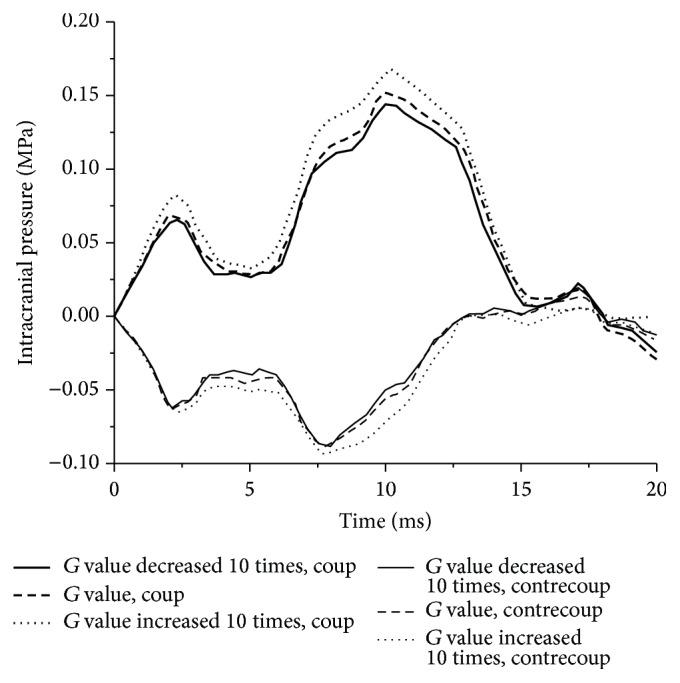
Intracranial pressure time histories with different *G* values.

The absolute values of coup and contrecoup pressure rise with the increase of *G* value as shown in Figure 5, which is plotted from the results of baseline, Ex_1, and Ex_2 experiments. The coup pressure increases from 155.1 kPa to 180.8 kPa while contrecoup pressure increases from −93.4 kPa to −100.5 kPa when *G*_0_ value increases from 4.9 kPa to 490 kPa and *G*_*∞*_ value increases from 1.62 kPa to 162 kPa.

#### 3.2.2. Shear Stress and Shear Strain

**Figure 6:**
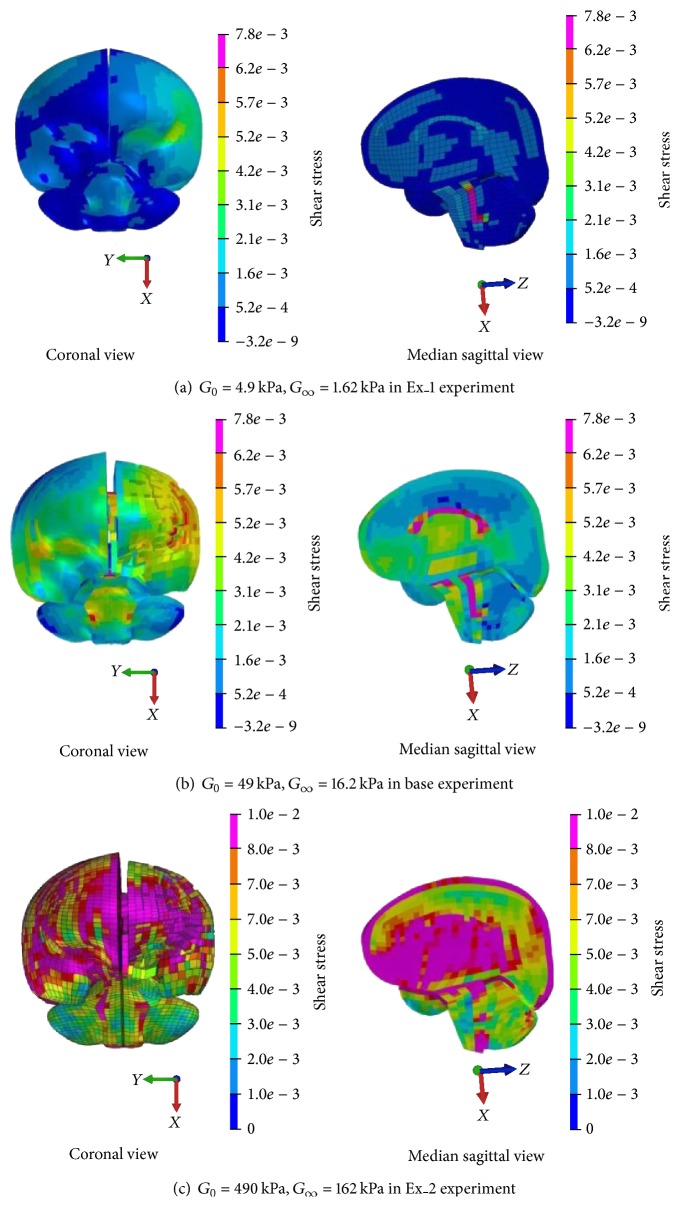
Shear stress distribution with different *G* values.

The impact direction of head in the simulation is not the same as the normal direction of engine hood surface. Therefore, the resultant head acceleration includes not only linear acceleration but also rotational acceleration during the impact process, which can lead to shear effect on brain tissues. Zhang et al. suggested that the probability of mild traumatic brain injury could be 50% when shear stress reaches 0.0078 MPa, and probability could be 80% when it exceeds 0.01 MPa [25]. If shear stress with 0.01 MPa is set as the injury criteria when evaluating the simulation results of brain tissues, it can be seen that the shear stress is higher than 0.01 MPa with an increase of *G* value by 10 times as shown in Figure 6(c). This indicates that risk of mild traumatic brain injury area is high. All the shear stresses illustrated in Figures 6(a) and 6(b) are less than 0.01 MPa when decreasing *G* value by 10 times, which indicates a lower risk of mild traumatic brain injury for brain tissues. If shear stress with 0.0078 MPa is set as injury criteria, injury risk occurs at mesencephalic aqueduct area of ventricle with 10 times lower *G* value (Figure 6(a)). Obviously, the risk of brain tissue injury increases with the increase of *G* value.

Likewise, peak shear stress also increases continuously with the increase of *G* value. When the *G* value is reduced by 10 times, the peak shear stress is 0.011 MPa and occurs at the dorsal pontine of brain stem. When the *G* value increases to 10 times, the peak shear stress reaches 0.054 MPa and occurs at the lateral parietal cortex in the impact side. Therefore, both peak values and peak areas of shear stress constantly vary with the increase of *G* value.

**Figure 7:**
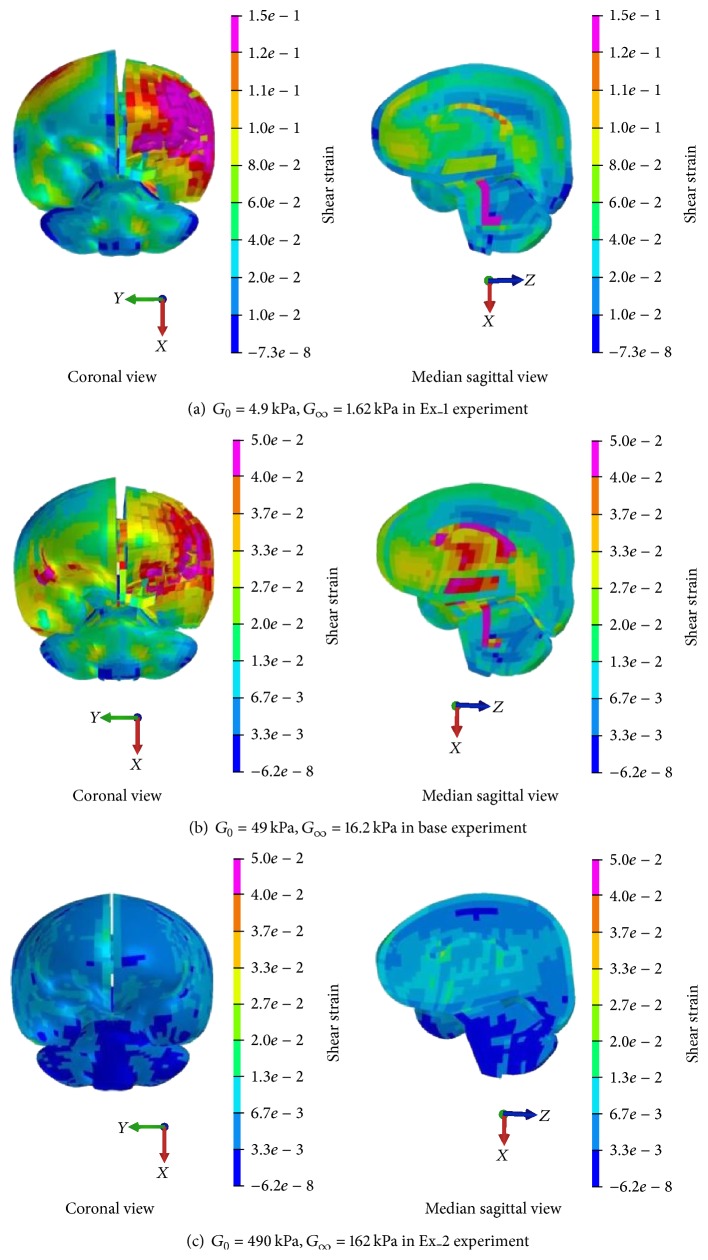
Shear strain distribution with different *G* values.

However, shear strain of brain tissues decreases with the increase of *G* value (Figure 7), whose trend is opposite to that of shear stress. The peak shear strain decreases from 0.20 to 0.012 when the *G*_0_ value increases from 4.9 kPa to 490 kPa and *G*_*∞*_ value increases from 1.62 kPa to 162 kPa. Also, the peak shear strain area occurs at temporal lobe with the 10 times decreased *G* value while it happened at corpus callosum in impact side with the 10 times increased *G* value.

### 3.3. Intracranial Response with Different *β* Values

#### 3.3.1. Intracranial Pressure

**Figure 8:**
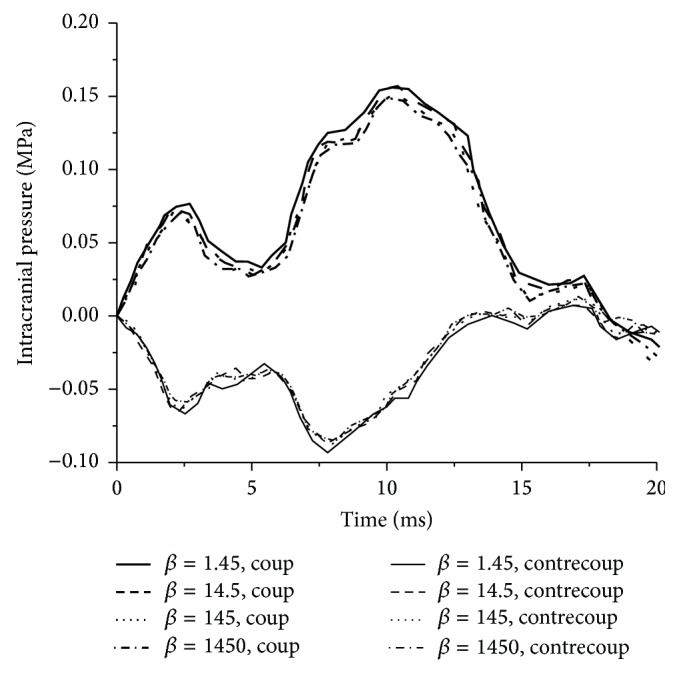
Intracranial pressure time histories with different *β* values.

Simulation results in Figure 8 from base, Ex_3, Ex_4, and Ex_5 experiments show that intracranial pressure time history curves with different decay coefficient *β* values almost overlap each other, which indicates that *β* variation has no effect on intracranial pressure. The peak coup pressures with different *β* values are 0.159 MPa.

#### 3.3.2. Shear Stress and Shear Strain

**Figure 9:**
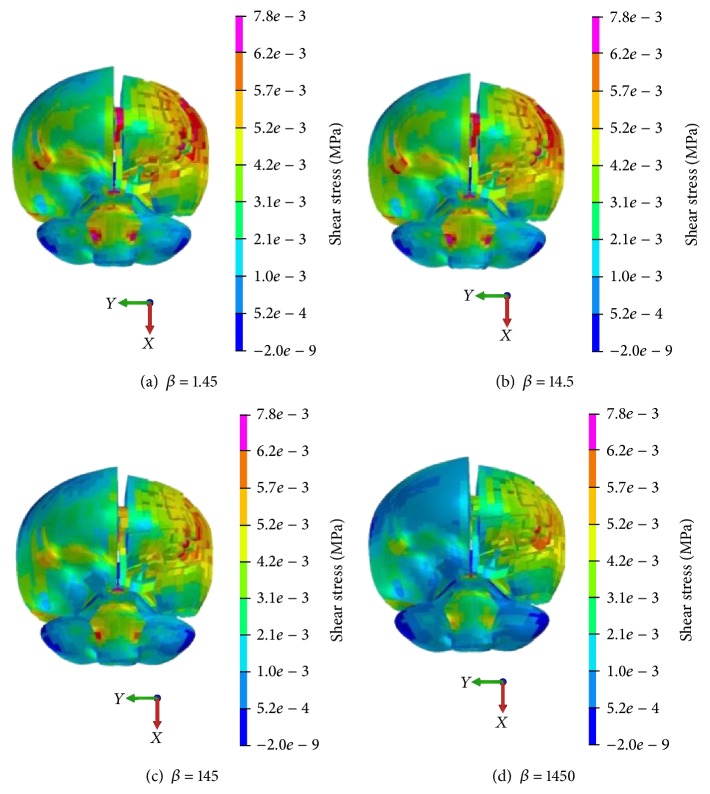
Brain shear stress distribution with different *β* value (coronal view).

**Figure 10:**
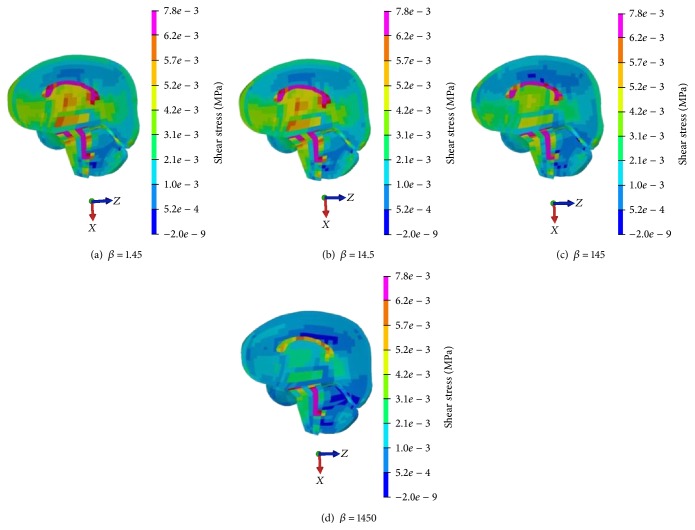
Brain shear stress distribution with different *β* values (sagittal view).

According to Zhang's shear stress injury criteria with 0.0078 MPa [25], brain shear stress distribution with different *β* values is shown in Figures 9 and 10. It can be seen that the injury risk area, especially at the lobe of cerebrum parietal cortex, ventrolateral pons of brain stem, and corpus callosum on impact side, decreases with the increase of *β* value.

**Figure 11:**
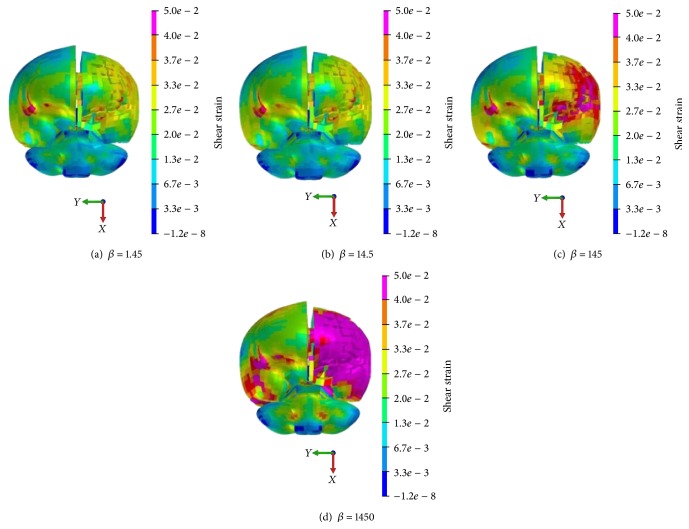
Brain shear strain distribution with different *β* value.

The upper limit values of brain shear strain with different *β* values were investigated in order to obtain the same high strain area at the cerebral cortex in the impact side. In Figure 11, it can be seen that the upper limit shear strain value is 0.055 with *β* value of 1.45 s^−1^ while it is 0.12 with *β* value of 1450 s^−1^.

Though *β* variation has no effect on intracranial pressure, it really has large effect on shear stress and shear strain. The brain injury risk is higher when the *β* value is 1450 s^−1^.

## 4. Conclusions

The finite element head model of a child with detailed anatomical structures was established based on CT images of a 6-year-old healthy child head. According to Euro-NCAP testing regulation, impact simulation experiments between the child head model and engine hood were studied. The influence of brain mechanical properties on the intracranial response was analyzed systematically through a comprehensive parametric study.

Intracranial pressure and shear stress of brain tissues increase with the increase of bulk modulus *K* while shear strain decreases. Likewise, values of peak shear stress and shear strain are both different with the variation of the *K* values.

The effects of shear modulus *G* on the variation of intracranial pressure, shear stress, and shear strain have the same trend as the bulk modulus *K*.

As for decay coefficient *β*, various *β* values almost have no influence on intracranial pressure. However, the peak shear stress decreases and shear strain increases with greater *β* value.

## Figures and Tables

**Table 1:** Brain mechanical properties found in the literature and used in the simulation.

Literature	*G*_0_ (kPa)	*G*_∞_ (kPa)	β (s^−1^)	*K* (MPa)
Roth et al. [9]	5.99	2.32	0.09248	2110
Nicolle et al. [16]	9.884	3.725	920	1125
Shuck and Advani [17]	49	16.2	145	1125
Lee [18]	26.9–110	2.87	50	1.25–5.44
DiMasi et al. [19]	34.474	17.23	100	68.948
Ruan [20]	528	168	35	127.9

In the table, *K* is bulk modulus.

**Table 2:** Material properties used in the head model.

	Density/kg·m^−3^	Poisson's ratio	*E*/MPa
Meninges	1140	0.45	31.5
CSF	1040	0.49	0.012
Scalp	1200	0.42	16.7
Cortical bone of skull	2150	0.22	9870
Cancellous bone of skull	2150	0.22	3690
Sutures	2150	0.22	1100

**Table 3:** Detailed experiment series with different parameters.

	*G*_0_ (kPa)	*G*_*∞*_ (kPa)	*β* (s^−1^)	*K* (MPa)
Base	49	16.2	145	2190
Ex_1	4.9	1.62	145	2190
Ex_2	490	162	145	2190
Ex_3	49	16.2	1.45	2190
Ex_4	49	16.2	14.5	2190
Ex_5	49	16.2	1450	2190
Ex_6	4.9	1.62	145	219
Ex_7	4.9	1.62	145	21.9
